# Developing a symptoms-based risk score for infectious syphilis among men who have sex with men

**DOI:** 10.1136/sextrans-2022-055550

**Published:** 2022-11-18

**Authors:** Silvia Achia Nieuwenburg, Elske Hoornenborg, Udi Davidovich, Henry John Christiaan de Vries, Maarten Schim van der Loeff

**Affiliations:** 1 Department of Infectious Diseases, Public Health Service Amsterdam, Amsterdam, The Netherlands; 2 Amsterdam Institute for Infection and Immunity (AII), Amsterdam UMC location University of Amsterdam, Amsterdam, The Netherlands; 3 Department of Dermatology, Amsterdam UMC location University of Amsterdam, Amsterdam, The Netherlands; 4 Department of Internal Medicine, Division of Infectious Diseases, Amsterdam UMC location University of Amsterdam, Amsterdam, the Netherlands

**Keywords:** syphilis, diagnosis, homosexuality, male

## Abstract

**Background:**

Syphilis incidence is rising among men who have sex with men (MSM). An online tool based on a risk score identifying men with higher risk of infectious syphilis could motivate MSM to seek care. We aimed therefore to develop a symptoms-based risk score for infectious syphilis.

**Methods:**

We included data from all consultations by MSM attending the Amsterdam Centre for Sexual Health in 2018–2019. Infectious syphilis (ie, primary, secondary or early latent syphilis) was diagnosed according to the centre’s routine protocol. Associations between symptoms and infectious syphilis were expressed as odds ratios (OR), with 95% confidence intervals (CI). Based on multivariable logistic regression models, we created risk scores, combining various symptoms. We assessed the area under the curve (AUC) and cut-off based on the Youden Index. We estimated which percentage of MSM should be tested based on a positive risk score and which percentage of infectious syphilis cases would then be missed.

**Results:**

We included 21,646 consultations with 11,594 unique persons. The median age was 34 years (IQR 27–45), and 14% were HIV positive (93% on antiretroviral treatment). We diagnosed 538 cases of infectious syphilis. Associations with syphilis symptoms/signs were strong and highly significant, for example, OR for a painless penile ulcer was 35.0 (CI 24.9 to 49.2) and OR for non-itching rash 57.8 (CI 36.8 to 90.9). Yet, none of the individual symptoms or signs had an AUC >0.55. The AUC of risk scores combining various symptoms varied from 0.68 to 0.69. For all risk scores using cut-offs based on Youden Index, syphilis screening would be recommended in 6% of MSM, and 59% of infectious syphilis cases would be missed.

**Conclusion:**

Symptoms-based risk scores for infectious syphilis perform poorly and cannot be recommended to select MSM for syphilis screening. All MSM with relevant sexual exposure should be regularly tested for syphilis.

**WHAT IS ALREADY KNOWN ON THIS TOPIC**

- A symptoms-based risk score for infectious syphilis motivating men who have sex with men (MSM) to seek care has not previously been developed.

**WHAT THIS STUDY ADDS**

- We developed a risk score based on symptoms and notification for syphilis. Individual symptoms included in the risk score were strongly associated with infectious syphilis, but the risk score had poor sensitivity and specificity.

**HOW THIS STUDY MIGHT AFFECT RESEARCH, PRACTICE OR POLICY**

- Symptoms-based risk scores for infectious syphilis cannot be recommended to select MSM for syphilis screening; all MSM at risk of syphilis should be regularly screened.

## Introduction

Syphilis, caused by the bacterium *Treponema pallidum*, remains a public health concern. In many countries, the incidence of syphilis is rising, especially among populations of men who have sex with men (MSM).^1^ If left untreated, it can lead to serious cardiovascular and neurological complications.^2^ Syphilis can also facilitate the transmission of HIV.^3^ It is highly contagious through sexual contact during the primary and secondary stage, as well as in the early latent stage. Long-acting penicillin G, an effective and inexpensive antibiotic, remains the recommended treatment for syphilis.^4^

The signs and symptoms of syphilis vary depending on the stage, and for this reason syphilis is also known as ‘the great imitator’.^5^ This makes it challenging for patients to recognise the disease and seek a health provider when they are symptomatic. The primary stage is characterised by a usually painless ulcer at the site of infection, usually appearing within 3 months after acquisition and disappearing within 6 weeks. If left untreated, the disease may progress to the secondary stage characterised by a non-itching rash.^6^ Early latent stage syphilis is asymptomatic and can be diagnosed with serological tests in patients without a history of primary or secondary symptoms.

The increasing incidence of syphilis calls for preventive efforts to reduce exposure to the infection. To reduce risk of ongoing transmission (especially in the primary and secondary stage) and of sequelae, early diagnosis and treatment are key, and it would be helpful if patients would be able to recognise the disease and be motivated to seek care.

Therefore, the Centre for Sexual Health of the Public Health Service of Amsterdam and the national non-governmental organisation STI-AIDS Netherlands launched a campaign to create awareness of syphilis in Amsterdam, the Netherlands. The awareness campaign, which ran from 2018 to 2019, included an online tool to enable individuals to self-identify symptoms that could be related to syphilis, and if indicated motivate them to seek care (testing and immediate treatment in case a diagnosis was made). This online tool included an expert-based algorithm of various symptoms; we also included a partner notification for syphilis in the risk score.^7^ The aim of the current study was to assess whether an evidence-based symptoms-based risk score could be developed, which could help individuals to self-identify possible infectious syphilis.

## Methods

There are few data from the peer-reviewed literature on the associations of symptoms with a current syphilis infection. We therefore developed a syphilis assessment algorithm in consultation with experts (dermatologists and internist-infectiologist). Based on the expert opinions, the following signs and symptoms were included in the risk algorithm: a painful or painless ulcer on the penis, in the mouth, at the anus or on the skin, with or without palpable regional lymph nodes, and an itching or non-itching rash with or without influenza-like symptoms. In addition, we also added partner notification for syphilis. A complete overview of the included items is provided in online supplemental table 1.

10.1136/sextrans-2022-055550.supp1 Supplementary data

To assess the validity of the expert-based algorithm, we used data from all consultations by MSM attending the Centre for Sexual Health of the Amsterdam Public Health Service, the Netherlands, from July 2018 to July 2019, regardless of reason of visit. Consultations were at the client’s own initiative and free of charge.

For this study, the outcome of interest was infectious syphilis, which comprises primary, secondary and early latent syphilis. A syphilis diagnosis was made according to the centre’s routine protocol using a combination of clinical signs and symptoms and laboratory testing based on the European guidelines for syphilis management.^8^ Serological screening for syphilis consisted of a treponemal test chemiluminescence immunoassay (CLIA, DiaSorin, Saluggia, Italy) confirmed by an immunoblot (INNO-LIA Syphilis, Innogenetics, Ghent, Belgium) and a non-treponemal test, the rapid plasma reagin (RPR, RPR NOSTICON II, bioMérieux, Marcy l’Etoile, France). In persons with suspected primary syphilis, we additionally performed dark-field microscopy of ulcer exudate and a PCR of ulcer scraping.^9^ Secondary syphilis was diagnosed if a client had a positive serology with characteristic skin or mucocutaneous lesions. Early latent syphilis was defined as confirmed positive serology without clinical manifestations, according to the European guidelines.^8^

We described person characteristics by medians and interquartile ranges (IQRs), and numbers and percentages. Univariable logistic regression was done using Generalized Estimating Equations (GEE), because of correlated data (multiple visits by the same person). Logistic regression was done to obtain ORs with 95% CIs for infectious syphilis diagnosis for each symptom and risk factor. Various multivariable models were constructed to enable creation of a series of prediction models and prediction scores. Our initial risk score A1 was based on the symptoms of the expert-based algorithm: ulcer in the mouth, ulcer on the skin, painless ulcer at the anus with palpable regional lymph nodes, painless ulcer on the penis with palpable regional lymph nodes, painless ulcer at the anus without palpable regional lymph nodes, painless ulcer on the penis without palpable regional lymph nodes, painful ulcer at the anus with palpable regional lymph nodes, painful ulcer on the penis with palpable regional lymph nodes, painful ulcer at the anus without palpable regional lymph nodes, painful ulcer on the penis without palpable regional lymph nodes, rash on the palms of the hands or soles of the feet, itching rash, non-itching rash and a partner notification for syphilis (online supplemental table 1).

In risk score A1, each factor was awarded 1 point if present and 0 if not, that is, without weighing. Risk score A2 included the same factors as risk score A1, but here each symptom/factor was given a weight equal to the rounded regression coefficient of the multivariable analysis. Risk score B1 was a simplified risk score. Here we only included the symptoms and risk factor (a partner notification for syphilis) that were significantly associated with infectious syphilis in model A1, without weighing. Risk score B2 included the same factors as risk score B1, but with weighing based on the regression coefficients. To optimise the risk scores, we also calculated risk scores C1 and C2. Penile ulcers were associated with infectious syphilis, independently of lymph nodes being palpable or not. Therefore, risk score C1 included the same factors as risk score B1, but rather than including painless and painful ulcers with and without regional palpable lymph nodes, we included painless and painful ulcers, both regardless of lymph nodes. Risk score C2 included the same factors as risk score C1, but with weighing based on the regression coefficients.

For each risk score, we assessed the area under the curve (AUC). To evaluate the clinical relevance of the risk scores A, B and C, the post-test probability of disease was evaluated. The positive predictive value of having infectious syphilis when having a score above the cut-off was estimated; the cut-off was based on the Youden Index. The Youden Index measures the effectiveness of a diagnostic marker as follows: sensitivity+specificity–1.^10^ As there is no generally agreed optimum of sensitivity and specificity combination, we selected the highest value for the Youden Index as optimal cut-off. Furthermore, we estimated which percentage of infectious syphilis cases would be missed, if patients would only be tested for syphilis if their score exceeded the cut-off of the risk score. A p value of <0.05 was considered statistically significant. We carried out analyses using Stata (V.15.1, StataCorp, College Station, Texas, USA).

## Results

In total, 21,646 consultations of 11,594 persons were included in this study (see table 1 which provides details of persons at their first visit in the study period). The median age was 34 years. A total of 1,586 (13.7%) were living with HIV; of the 1,501 with information on viral load, 1,379 (91.9%) reported an undetectable viral load. In total, 421 (3.6%) persons were notified for syphilis.

**Table 1:** Characteristics of all MSM at first consultation in study period; Amsterdam Centre for Sexual Health, July 2018–July 2019

Variables	Total (n=11,594)	Infectious syphilis (n=274)	No infectious syphilis (n=11,320)	P value
Age in years (median, IQR)	34 (27–45)	36 (29–47)	34 (27–45)	0.009
Country of birth, n (%)				
Netherlands	7,332 (63.2)	155 (56.6)	7,177 (63.4)	0.020
Othe4,r	4,262 (36.8)	119 (43.4)	4,143 (36.6)	
Sexual behaviour, n (%)				
MSM	10,237 (88.3)	260 (94.9)	9,977 (88.1)	0.001
MSMW	1,357 (11.7)	14 (5.1)	1,343 (11.9)	
HIV status, n (%)				
Negative	10,005 (86.3)	152 (55.5)	9,853(87.0)	<0.001
Positive	1,586 (13.7)	122 (44.5)	1,464 (12.9)	
Unknown	3 (0.0)	3 (0.0)	0 (0.0)	
On ART, n (%)*	1,396 (93.0)	103 (89.6)	1,293 (93.3)	0.132
HIV VL as reported by patient, n (%)				
Undetectable	1,379 (86.9)	105 (86.1)	1,274 (87.0)	<0.001
Detectable	33 (2.1)	3 (2.5)	30 (2.0)	
Notified for syphilis	421 (3.6)	56 (20.4)	365 (3.2)	<0.001
STD diagnosis at current visit, n (%)				
Chlamydia	1,146 (9.9)	52 (19.0)	1,094 (9.7)	<0.001
Gonorrhoea	1,290 (11.1)	62 (22.6)	1,228 (10.9)	<0.001
Lymphogranuloma venereum	73 (0.6)	8 (2.9)	65 (0.6)	<0.001
Hepatitis B	9 (0.1)	1 (0.4)	8 (0.1)	0.084
Hepatitis C	3 (0)	0 (0)	3 (0)	0.788

*For those who are HIV positive, data on ART use of 85 participants were missing.

ART, antiretroviral therapy; IQR, interquartile range; MSM, men who have sex with men; MSMW, men who have sex with men and women; STD, sexually transmitted disease; VL, viral load.

At the 21,646 consultations, 538 cases of infectious syphilis were diagnosed (2.5%). A total of 1270 men (5.9%) reported at least one of the symptoms included in the expert-based risk score. In 260 consultations (1.2%), a penile ulcer was present, in 114 (0.5%) an anal ulcer and in 148 (0.7%) a skin rash. The characteristic rash on hand palms or feet soles was rare, present in 23 (0.1%) consultations.

In univariable analysis, all symptoms and the risk factor (a partner notification for syphilis) used were associated with a diagnosis of infectious syphilis (online supplemental table 2). The self-reported symptoms: non-itching rash with influenza-like symptoms (OR 86.8, 95% CI 35.2 to 214.1), rash on the soles of the feet (OR 78.6, 95% CI 7.1 to 867.6), painless anal ulcer with palpable regional lymph nodes (OR 78.6, 95% CI 7.1 to 867.6) and painless penile ulcer with palpable regional lymph nodes (OR 76.6, 95% CI 34.0 to 172.6) were most strongly associated with an infectious syphilis diagnosis. The AUCs of the individual symptoms or signs were between 0.50 and 0.55.

In a multivariable analysis including all symptoms (and a partner notification for syphilis) included in the expert-based risk score (model A), the following symptoms were strongly associated with an infectious syphilis diagnosis: painless penile ulcer with palpable regional lymph nodes (adjusted OR (aOR) 85.8, 95% CI 36.6 to 201.0), painless anal ulcer with palpable regional lymph nodes (aOR 63.8, 95% CI 4.1 to 991.8) and non-itching rash (aOR 54.3, 95% CI 32.7 to 90.2) (table 2). In model B, which is similar to model A, but with non-significant symptoms omitted, the same symptoms were strongly associated with an infectious syphilis diagnosis: painless penile ulcer with palpable regional lymph nodes (aOR 85.7, 95% CI 36.6 to 200.6), non-itching rash (aOR 64.8, 95% CI 40.4 to 103.7) and painless anal ulcer with palpable regional lymph nodes (aOR 63.6, 95% CI 4.1 to 989.3). Because a painless penile ulcer was strongly associated with infectious syphilis regardless of palpable regional lymph nodes, and also a painful penile ulcer was strongly associated with infectious syphilis regardless of palpable regional lymph nodes, we made model C in which ‘painless penile ulcer’ and ‘painful penile ulcer’ were included rather than the four separate items. In this optimised model C, a painless anal ulcer with inguinal lymph nodes (aOR 65.4, 95% CI 4.4 to 981.1), non-itching rash (aOR 64.8, 95% CI 40.5 to 103.7) and painless penile ulcer (aOR 36.1, 95% CI 25.2 to 51.6) were most strongly associated with an infectious syphilis diagnosis.

**Table 2:** Multivariable analysis of possible syphilis symptoms and their association with a diagnosis of infectious syphilis among MSM; Amsterdam Centre for Sexual Health, 2018–2019

Coding	Symptoms/risk factor		Model A* aOR (95% CI)	βˆ	Model B† aOR (95% CI)	βˆ	Model C‡ aOR (95% CI)	βˆ
1.m	Ulcer in the mouth	No	Ref					
		Yes	3.2 (0.9 to 12.0)	1.2				
1.s	Ulcer on the skin§	No	Ref					
		Yes	0.7 (0.1 to 4.4)	−0.3				
1.1.p	Painless ulcer on the penis	No					Ref	
		Yes					36.1 (25.2 to 51.6)	3.6
1.1.1.a	Painless ulcer at the anus with lymph nodes¶	No	Ref		Ref		Ref	
		Yes	63.8 (4.1 to 991.8)	4.2	63.6 (4.1 to 989.3)	4.2	65.4 (4.4 to 981.1)	4.2
1.1.1.p	Painless ulcer on the penis with lymph nodes	No	Ref		Ref			
		Yes	85.8 (36.6 to 201.0)	4.5	85.7 (36.6 to 200.6)	4.5		
1.1.2.a	Painless ulcer at the anus without lymph nodes	No	Ref					
		Yes	1.4 (0.3 to 6.5)	0.4				
1.1.2.p	Painless ulcer on the penis without lymph nodes	No	Ref		Ref			
		Yes	29.1 (19.5 to 43.4)	3.7	30.0 (20.2 to 44.7)	3.4		
1.2.p	Painful ulcer on the penis	No					Ref	
		Yes					21.7 (14.2 to 33.2)	3.1
1.2.1.a	Painful ulcer at the anus with lymph nodes	No	Ref					
		Yes	1.5 (0.2 to 11.7)	0.4				
1.2.1.p	Painful ulcer on the penis with lymph nodes	No	Ref		Ref			
		Yes	11.5 (4.1 to 32.0)	2.4	13.0 (4.9 to 34.4)	2.6		
1.2.2.a	Painful ulcer at the anus without lymph nodes	No	Ref		Ref		Ref	
		Yes	9.6 (4.9 to 18.6)	2.3	9.6 (5.0 to 18.7)	2.3	9.7 (5.0 to 18.8)	2.3
1.2.2.p	Painful ulcer on the penis without lymph nodes	No	Ref		Ref			
		Yes	25.0 (15.6 to 39.9)	3.2	24.7 (15.5 to 39.6)	3.2		
2.paso	Rash on the palms of the hands or soles of the feet	No	Ref					
		Yes	2.6 (0.8 to 8.1)					
2.1	Itching rash¶	No	Ref		Ref		Ref	
		Yes	7.4 (3.4 to 16.3)	2.0	8.1 (3.8 to 17.2)	2.1	8.0 (3.7 to 17.0)	2.1
2.2	Non-itching rash	No	Ref		Ref		Ref	
		Yes	54.3 (32.7 to 90.2)	4.0	64.8 (40.4 to 103.7)	4.2	64.8 (40.5 to 103.7)	4.2
3.	Notified for syphilis	No	Ref		Ref		Ref	
		Yes	4.4 (3.3 to 5.7)	1.5	4.4 (3.3 to 5.7)	1.5	4.3 (3.3 to 5.7)	1.5

β∧ is the regression coefficient of the logistic regression models (i.e. the log of the Odds Ratio).

*Model A is based on the symptoms of the expert-based algorithm: ulcer in the mouth, ulcer on the skin, painless ulcer at the anus with palpable regional lymph nodes, painless ulcer on the penis with palpable regional lymph nodes, painless ulcer at the anus without palpable regional lymph nodes, painless ulcer on the penis without palpable regional lymph nodes, painful ulcer at the anus with palpable regional lymph nodes, painful ulcer on the penis with palpable regional lymph nodes, painful ulcer at the anus without palpable regional lymph nodes, painful ulcer on the penis without palpable regional lymph nodes, rash on the palms of the hands or soles of the feet, itching rash, non-itching rash and a partner notification for syphilis.

†Model B is based on the symptoms and risk factor (a partner notification for syphilis) that were significantly associated with infectious syphilis: painless ulcer at the anus with palpable regional lymph nodes, painful ulcer at the anus without palpable regional lymph nodes, painless ulcer on the penis with palpable regional lymph nodes, painless ulcer on the penis without palpable regional lymph nodes, painful ulcer on the penis with palpable regional lymph nodes, painful ulcer on the penis without palpable regional lymph nodes, itching rash, non-itching rash and a partner notification for syphilis.

‡Model C is based on model B, but simplified by including painless and painful ulcers (both regardless of lymph nodes): painless ulcer at the anus with palpable regional lymph nodes, painful ulcer at the anus without palpable regional lymph nodes, painless ulcer on the penis, painful ulcer on the penis, itching rash, non-itching rash and a partner notification for syphilis.

§Skin is defined as location other than anogenital (anus or penis) or mouth.

¶Rash associated with syphilis: a maculopapular exanthema or erythematous exanthema.

aOR, adjusted OR; MSM, men who have sex with men.

**Figure 1:**
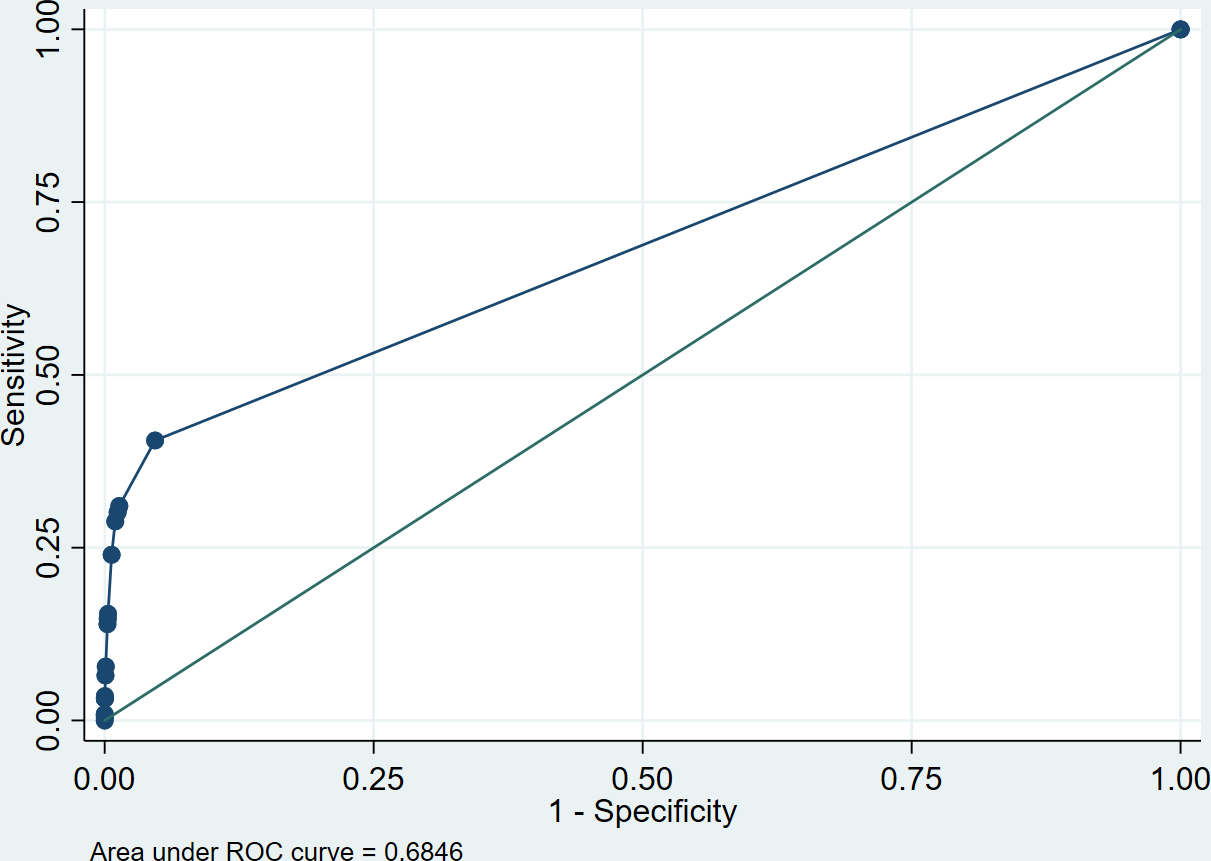
ROC curve of score C2 among MSM with infectious syphilis; Amsterdam Centre for Sexual Health. Risk score C2 is comprised of the following symptoms (between brackets the weights of each symptom): painless anal ulcer with palpable regional lymph nodes (4.2)+painful anal ulcer with no palpable regional lymph nodes (2.3)+painless penile ulcer (3.5)+painful penile ulcer (3.1)+itching rash (2.1)+non-itching rash (4.2)+a partner notification for syphilis (1.5). MSM, men who have sex with men; ROC, receiver operating characteristics.

Risk score A1, based on the expert-based algorithm, had a sensitivity of 40.9% and a specificity of 95.0%. Based on the cut-off of 1.0 (based on Youden Index), 5.9% of men would have to be tested and 59% of syphilis cases would remain undetected. The risk score using weighing (A2) performed similarly. The other four risk scores (B1, B2, C1, C2) performed almost identical to the risk scores A1 and A2. The AUC was 0.68 for risk score A1 based on the expert-opinion algorithm (figure 1). The AUCs of the other risk scores, including our optimised risk score C2, were all also 0.68. Table 3 shows the sensitivity and specificity of the various risk scores for infectious syphilis.

**Table 3:** Performance of several risk scores for infectious syphilis among MSM; Amsterdam Centre for Sexual Health, July 2018–July 2019

Risk score*	Cut-off†	Infectious syphilis among those with a score of at least the cut-off	Infectious syphilis among those with a score below the cut-off	Sensitivity (95% CI)	Specificity (95% CI)	AUC	% to be tested
Score A1	1.0	220/1,270	318/20,376	40.9 (36.7 to 45.2)	95.0 (94.7 to 95.3)	0.6811	5.9
Score A2	0.9	220/1,226	318/20,420	40.9 (36.7 to 45.2)	95.2 (94.9 to 95.5)	0.6846	5.7
Score B1	1.0	218/1,206	320/20,440	40.5 (36.3 to 44.8)	95.3 (95.0 to 95.6)	0.6811	5.6
Score B2	1.5	218/1,206	320/20,440	40.5 (36.3 to 44.8)	95.3 (95.0 to 95.6)	0.6846	5.6
Score C1	1.0	218/1,206	320/20,440	40.5 (36.3 to 44.8)	95.3 (95.0 to 95.6)	0.6811	5.6
Score C2	1.5	218/1,206	320/20,440	40.5 (36.3 to 44.8)	95.3 (95.0 to 95.6)	0.6846	5.6

*Risk scores are based on the multivariable models as explained in table 2. Risk scores A1, B1 and C1 are based on a summation of all risk factors, each with identical weight (one). Risk scores A2, B2 and C2 are based on a summation of the weights of all risk factors, the weight being identical to the regression coefficient of a factor in the multivariable model (see table 2).

†Based on the Youden Index.

AUC, area under the curve; MSM, men who have sex with men.

## Discussion

We aimed to assess whether an evidence-based symptoms-based risk score could be developed to identify individuals with infectious syphilis. The optimised risk score C2, including six symptoms and a partner notification for syphilis, had a sensitivity of 40.5% (95% CI 36.3% to 44.8%), a specificity of 95.3% (95% CI 95.0% to 95.6%) and an AUC of 0.68. Based on this score, 5.6% of the study population would be eligible for syphilis testing and 41% of the cases would be identified.

We had aimed that a symptoms-based risk score would enable MSM to self-identify possible infectious syphilis to increase early testing and diagnosis, and avoid further transmission and complications. To our knowledge, there are no studies on the development of a symptoms-based risk score for infectious syphilis. One study developed a simple-to-use nomogram to predict the risk of syphilis, and several other studies described the risk factors for syphilis based on sociodemographic, clinical or behavioural data.^11–14^ The main objective of the current study was to develop a symptoms-based risk score so that individuals would seek help when symptomatic, analogous to similar algorithms for acute HIV infection. Several symptom and behavioural risk scores for acute HIV have been developed and validated successfully.^15–18^ The symptoms-based risk scores may have the advantage above behavioural scores as they may be generalisable among different populations and be unaffected by frequency of high-risk behaviour. Unfortunately, our risk scores did not have promising characteristics and infections would be missed by using the tool. Various iterations to increase sensitivity or specificity did not lead to improved performance; this is probably due to the fact that many infectious syphilis cases are asymptomatic.

This study has several strengths. The data of the Centre for Sexual Health of Amsterdam provided us with a large sample size. Secondly, self-reported symptoms were systematically noted in all the consultations. Furthermore, all clients were tested systematically based on their symptoms and risk behaviour. All assessed symptoms in this study were highly predictive for infectious syphilis. The study also has several limitations. We chose not to include risk behaviour in the risk score. Including risk behaviour might improve the algorithm, yet this would not benefit our objective of self-reported symptoms-based algorithms leading to seeking care. Furthermore, the cut-offs of our risk scores were based on the Youden Index, but this is a rather arbitrary cut-off; the optimal cut-off may depend on the prevalence of infectious syphilis in the population, financial resources and acceptability of low sensitivity. Lastly, we used data of MSM visiting the Centre for Sexual Health; possible results may not be generalisable to all MSM including those who do not yet seek sexual healthcare.

For the prevention of syphilis, multiple approaches are necessary. An awareness campaign with a symptoms-based risk score will likely not prevent the ongoing transmission of infectious syphilis. Early identification of the infection and timely treatment are helpful to reduce the duration of infection and onwards sequela. An online symptoms tool might in some cases be harmful as it may warrant incorrect assessment of no risk. Thus, all MSM who engage in sexual behaviour that places them at higher risk of acquiring STIs should be regularly tested for syphilis.^8^ Yet, an online tool might help to motivate MSM to seek help timely in case of suspected symptoms.

In conclusion, our proposed symptoms-based risk scores performed poorly to diagnose infectious syphilis. Thus, we cannot recommend a symptoms-based risk score to select MSM for syphilis screening. All MSM with relevant sexual exposure should be regularly tested for syphilis.

## Data Availability

All data relevant to the study are included in the article or uploaded as supplemental information.
