# Supplementary material for: Developing a symptoms-based risk score for infectious syphilis among men who have sex with men
Source: Sex Transm Infect. 2022 Nov 18;99(5):324–9. doi: 10.1136/sextrans-2022-055550 (PMC10359546; doi:10.1136/sextrans-2022-055550)
Supplement: Supplementary data [file sextrans-2022-055550supp001.pdf]

Supplementary file

Developing a symptom-based risk score for infectious syphilis among men who have sex with men

Nieuwenburg S.A.[1],Hoornenborg E[1],Davidovich U.[1], de Vries H.J.C.[1,2,3], Schim van der Loeff M.F.[1,2,4]

[1] Department of Infectious Diseases, Public Health Service Amsterdam, Amsterdam, the Netherlands

[2] Amsterdam UMC location University of Amsterdam, Amsterdam Institute for Infection and Immunity (AII), Meibergdreef 9, Amsterdam, the Netherlands

[3] Amsterdam UMC location University of Amsterdam, Department of Dermatology, Meibergdreef 9, Amsterdam, the Netherlands

[4] Amsterdam UMC location University of Amsterdam, Department of Internal Medicine, Division of Infectious Diseases, Meibergdreef 9, Amsterdam, the Netherlands

Nieuwenburg SA, *et al. Sex Transm Infect* 2023; 99:324–329. doi: 10.1136/sextrans-2022-055550

Supplementary Table 1 Symptoms and risk factor assessed for the association with infectious syphilis among MSM. Amsterdam Centre for Sexual Health, 2018-2019. The table lists all symptoms and combinations of symptoms that were evaluated in the univariable analysis; the columns for each risk score indicate which factors were included in the multivariable model underpinning that risk score.

Coding	Symptoms/risk factor	Included in Score A*	Included in Score B**	Included in Score C***
1.	Ulcer[a]			
1.a	at the anus			
1.p	on the penis			
1.m	in the mouth	x		
1.s	on the skin[b]	x		
1.1	Painless ulcer			
1.1.a	at the anus			
1.1.p	on the penis			x
1.1.m	in the mouth			
1.1.s	on the skin			
1.1.1	Painless ulcer with lymph nodes[c]			
1.1.1.a	at the anus	x	x	x
1.1.1.p	on the penis	x	x	
1.1.1.m	in the mouth			
1.1.1.s	on the skin			
1.1.2	Painless ulcer without lymph nodes			
1.1.2a	at the anus	x		
1.1.2.p	on the penis	x	x	
1.1.2.m	in the mouth			
1.1.2.s	on the skin			
1.2	Painful ulcer			
1.2.a	at the anus			
1.2.p	on the penis			x
1.2.m	in the mouth			
1.2.s	on the skin			

1.2.1	Painful ulcer with lymph nodes			
1.2.1.a	at the anus	x		
1.2.1.p	on the penis	x	x	
1.2.1.m	in the mouth			
1.2.1.s	on the skin			
1.2.2	Painful ulcer without lymph nodes			
1.2.2.a	at the anus	x	x	x
1.2.2.p	on the penis	x	x	
1.2.2.m	in the mouth			
1.2.2.s	on the skin			
2.	Rash [d]			
2.pa	on the palms of the hands			
2.so	on the soles of the feet			
2.tr	on the trunk			
2.paso	on the palms of the hands or soles of the feet	x		
2.1	Itching rash	x	x	x
2.1.1	without flu-like symptoms			
2.1.2	with flu-like symptoms			
2.2	Non-itching rash	x	x	x
2.2.1	without flu-like symptoms			
2.2.2	with flu-like symptoms			
3.	Notified for syphilis	x	x	x

* Risk scores A1 and A2 contain the same set of symptoms and risk factor (partner notification for syphilis); in risk score A1 each factor has the same weight, and in risk score A2 each factor has a weight identical to the regression coefficient of that factor.
** Risk scores B1 and B2 contain the same set of symptoms and risk factor (partner notification for syphilis); in risk score B1 each factor has the same weight, and in risk score B2 each factor has a weight identical to the regression coefficient of that factor.
*** Risk scores C1 and C2 contain the same set of symptoms and risk factor (partner notification for syphilis); in risk score C1 each factor has the same weight, and in risk score C2 each factor has a weight identical to the regression coefficient of that factor.
[a] Ulcer is defined as having an ulcer at the anus, on the penis, in the mouth or on the skin
[b] Skin is defined as location other than anogenital (anus or penis) or mouth
[d] Rash associated with syphilis: a maculopapular exanthema or erythematous exanthema

Supplementary Table 2 Possible syphilis symptoms and risk factor and their association with a diagnosis of infectious syphilis among 21,646 consultations with MSM. Amsterdam STI outpatient clinic, 2018-2019.

Coding	Symptoms/risk factor*		Number with infectious syphilis among all clients in the category; n/N (%)	Crude Odds ratio (95% CI)	β**
1.	Ulcer[a]	No	419/21,252 (2.0%)	Ref	
		Yes	119/394 (30.2%)	21.5 (17.0-27.2)	3.1
1.a	at the anus	No	517/21,532 (2.4%)	Ref	
		Yes	21/114 (18.4%)	9.2 (5.7-14.8)	2.2
1.p	on the penis	No	437/21,386 (2.0%)	Ref	
		Yes	101/260 (38.9%)	30.4 (23.3-39.7)	3.4
1.m	in the mouth	No	534/21,620 (2.5%)	Ref	
		Yes	4/26 (15.4%)	7.2 (2.5 -20.9)	2.0
1.s	on the skin[b]	No	536/21.626 (2.5%)	Ref	
		Yes	2/20 (10.0%)	4.4 (1.0-18.9)	1.5
1.1	Painless ulcer	No	472/21,647 (2.20%)	Ref	
		Yes	66/179 (36.9%)	26.0 (18.9-35.6)	3.3
1.1.a	at the anus	No	533/21,614 (2.5%)	Ref	
		Yes	5/32 (15.6%)	7.3 (2.8-19.1)	2.0
1.1.p	on the penis	No	474/21,501 (2.2%)	Ref	
		Yes	64/145 (44.1%)	35.0 (24.9-49.2)	3.6
1.1.m	in the mouth	No	536/21,638 (2.5%)	Ref	
		Yes	2/8 (25.0%)	13.0 (2.6-64.8)	2.6
1.1.s	on the skin	No	537/21,638 (2.5%)	Ref	
		Yes	1/8 (12.5%)	5.6 (0.7-45.6)	1.7
1.1.1	Painless ulcer with lymph nodes	No	518/21,608 (2.4%)	Ref	
		Yes	20/38 (52.6%)	45.2 (23.8-86)	3.8
1.1.1.a	at anus	No	536/21,643 (2.5%)	Ref	
		Yes	2/3 (66.7%)	78.6 (7.1-867.6)	4.4

1.1.1.p	on the penis	No	521/21,620 (2.4%)	Ref	
		Yes	17/26 (65.4%)	76.6 (34.0-172.6)	4.3
1.1.1.m	in the mouth	No	536/21,644 (2.5%)		
		Yes	0/2 (0.0)	N/A[c]	N/A[c]
1.1.1.s	on the skin	No	538/21,645 (2.5%)		
		Yes	0/1 (0.0)	N/A[c]	N/A[c]
1.1.2	Painless ulcer without lymph nodes	No	490/21,497 (2.3%)	Ref	
		Yes	48/149 (32.2%)	20.4 (14.3-29.0)	3.0
1.1.2.a	at the anus	No	535/21,617 (2.5%)	Ref	
		Yes	3/29 (10.3%)	4.6 (1.4-15.1)	1.5
1.1.2.p	on the penis	No	491/21,527 (2.3%)	Ref	
		Yes	47/119 (39.5%)	27.9 (19.1-40.8)	3.3
1.1.2.m	in the mouth	No	536/21,640 (2.5%)	Ref	
		Yes	2/6 (33.3%)	19.5 (3.6-107.1)	3.0
1.1.2.s	on the skin	No	537/21,639 (2.5%)	Ref	
		Yes	1/7 (14.3%)	6.5 (0.8-54.3)	1.8
1.2	Painful ulcer	No	486/21,433 (2.3%)	Ref	
		Yes	52/213 (24.4%)	13.9 (10.1-19.3)	2.6
1.2.a	at the anus	No	523/21,565 (2.4%)	Ref	
		Yes	15/81 (18.5%)	9.1 (5.2-16.1)	2.2
1.2.p	on the penis	No	501/21,531 (2.3%)	Ref	
		Yes	37/115 (32.2%)	19.9 (13.3-29.7)	3.0
1.2.m	in the mouth	No	536/21,628 (2.5%)	Ref	
		Yes	2/18 (11.1%)	5.0 (1.1-21.5)	1.6
1.2.s	on the skin	No	538/21,635 (2.5%)		
		Yes	0/11(0.0%)	N/A[c]	N/A[c]
1.2.1	Painful ulcer with lymph nodes	No	528/21,599 (2.4%)	Ref	
		Yes	10/47 (21.3%)	10.8 (5.3-21.8)	2.4
1.2.1.a	at the anus	No	536/21,631 (2.5%)	Ref	
		Yes	2/15 (13.3%)	6.1 (1.4-27.0)	1.8
1.2.1.p	on the penis	No	532/21,621 (2.5%)	Ref	

		Yes	6/25 (24.0%)	12.5 (5.0-31.3)	2.5
1.2.1.m	in the mouth	No	536/21,638 (2.5%)	Ref	
		Yes	2/8 (25.0%)	13.1 (2.6-65.0)	2.6
1.2.1.s	on the skin	No	538/21,645 (2.5%)		
		Yes	0/1 (0.0%)	N/A[c]	N/A[c]
1.2.2	Painful ulcer without lymph nodes	No	496/21,478 (2.3%)	Ref	
		Yes	42/168 (25.0%)	14.1 (9.8-20.2)	2.6
1.2.2.a	at the anus	No	525/21,580 (2.4%)	Ref	
		Yes	13/66 (19.7%)	9.8 (5.3-18.1)	2.3
1.2.2.p	on the penis	No	507/21,556 (2.4%)	Ref	
		Yes	31/90 (34.4%)	21.8 (14.0-34)	3.1
1.2.2.m	in the mouth	No	538/21,636 (2.5%)		
		Yes	0/10 (0.0%)	N/A[c]	N/A[c]
1.2.2.s	on the skin	No	538/21,636 (2.5%)		
		Yes	0/10 (0.0%)	N/A[c]	N/A[c]
2.	Rash[d]	No	471/21,498 (2.2%)	Ref	
		Yes	67/148 (45.3%)	36.9 (26.4-51.7)	3.6
2.pa	on the palms of the hand	No	524/21,624 (2.4%)	Ref	
		Yes	14/22 (63.6%)	71.2 (29.7-170.6)	4.3
2.so	on the soles of the feet	No	536/21,634 (2.5%)	Ref	
		Yes	2/3 (66.7%)	78.6 (7.1-867.6)	4.4
2.tr	on the trunk	No	477/21,509 (2.2%)	Ref	
		Yes	61/137 (44.5%)	35.4 (24.9-50.1)	3.6
2.paso	on the palms of the hands or soles of the feet	No	523/21,623 (2.4%)	Ref	
		Yes	15/23 (65.2%)	76.4 (32.2-181.1)	4.3
2.1	Itching rash	No	526/21,593 (2.4%)	Ref	
		Yes	12/53 (22.6%)	11.7 (6.1-22.4)	2.5
2.1.1	without flu-like symptoms	No	526/21,593 (2.4%)	Ref	
		Yes	12/53 (22.6%)	11.7 (6.1-22.4)	2.5
2.1.2	with flu-like symptoms	No	538/21,646 (2.5%)		
		Yes	0/0 (0.0%)	N/A[c]	N/A[c]

2.2	Non-itching rash	No	492/21.566 (2.3%)	Ref	
		Yes	46/80 (57.5%)	57.8 (36.8-90.9)	4.1
2.2.1	without flu-like symptoms	No	507/21.588 (2.4%)	Ref	
		Yes	31/58 (53.5%)	47.7 (28.3-80.4)	3.9
2.2.2	with flu-like symptoms	No	523/21.624 (2.4%)	Ref	
		Yes	15/22 (68.2%)	86.8 (35.2-214.1)	4.5
3.	Having been notified for syphilis	No	442/20.818 (2.1%)	Ref	
		Yes	96/828 (11.6%)	6.1 (4.8-7.7)	1.8

[a] Ulcer is defined as having an ulcer at the anus, on the penis, in the mouth or on the skin. [b] Skin is defined as location other than anogenitalia (anus or penis) or mouth. [c]N/A= not applicable, because of the low numbers of observations. [d] Rash associated with syphilis: a maculopapular exanthema or erythematous exanthema

* This table is built up hierarchically. For example, a man with a painless ulcer on the penis with palpable regional lymph nodes and no other symptoms will appear in the "Yes" row of all of the following variables: ulcer (at the anus, on the penis, in the mouth, or on the skin), ulcer on the penis, painless ulcer (at the anus, on the penis, in the mouth, or on the skin), painless ulcer on the penis, painless ulcer (at the anus, on the penis, in the mouth, or on the skin) with palpable regional lymph nodes, painless ulcer on the penis with palpable regional lymph nodes. He will appear in the "No" row of all other variables.

** β is the regression coefficient of the logistic regression models (i.e. the log of the Odds Ratio).
